# The impact of migration and antimicrobial resistance on the transmission dynamics of typhoid fever in Kathmandu, Nepal: A mathematical modelling study

**DOI:** 10.1371/journal.pntd.0005547

**Published:** 2017-05-05

**Authors:** Neil J. Saad, Cayley C. Bowles, Bryan T. Grenfell, Buddha Basnyat, Amit Arjyal, Sabina Dongol, Abhilasha Karkey, Stephen Baker, Virginia E. Pitzer

**Affiliations:** 1 Department of Epidemiology of Microbial Diseases, Yale School of Public Health, Yale University, New Haven, Connecticut, United States of America; 2 David Geffen School of Medicine, University of California Los Angeles, Los Angeles, California, United States of America; 3 Department of Ecology and Evolutionary Biology, Princeton University, Princeton, New Jersey, United States of America; 4 Fogarty International Center, Bethesda, Maryland, United States of America; 5 Oxford University Clinical Research Unit, Patan Academy of Health Sciences, Kathmandu, Nepal; 6 Centre for Tropical Medicine and Global Health, University of Oxford, Oxford, United Kingdom; 7 The Hospital for Tropical Diseases, Wellcome Trust Major Overseas Programme, Oxford University Clinical Research Unit, Ho Chi Minh City, Vietnam; Georgia Southern University Jiann-Ping Hsu College of Public Health, UNITED STATES

## Abstract

**Background:**

A substantial proportion of the global burden of typhoid fever occurs in South Asia. Kathmandu, Nepal experienced a substantial increase in the number of typhoid fever cases (caused by *Salmonella* Typhi) between 2000 and 2003, which subsequently declined but to a higher endemic level than in 2000. This epidemic of *S*. Typhi coincided with an increase in organisms with reduced susceptibility against fluoroquinolones, the emergence of *S*. Typhi H58, and an increase in the migratory population in Kathmandu.

**Methods:**

We devised a mathematical model to investigate the potential epidemic drivers of typhoid in Kathmandu and fit this model to weekly data of *S*. Typhi cases between April 1997 and June 2011 and the age distribution of *S*. Typhi cases. We used this model to determine if the typhoid epidemic in Kathmandu was driven by heightened migration, the emergence of organisms with reduced susceptibility against fluoroquinolones or a combination of these factors.

**Results:**

Models allowing for the migration of susceptible individuals into Kathmandu alone or in combination with the emergence of *S*. Typhi with reduced susceptibility against fluoroquinolones provided a good fit for the data. The emergence of organisms with reduced susceptibility against fluoroquinolones organisms alone, either through an increase in disease duration or increased transmission, did not fully explain the pattern of *S*. Typhi infections.

**Conclusions:**

Our analysis is consistent with the hypothesis that the increase in typhoid fever in Kathmandu was associated with the migration of susceptible individuals into the city and aided by the emergence of reduced susceptibility against fluoroquinolones. These data support identifying and targeting migrant populations with typhoid immunization programmes to prevent transmission and disease.

## Author summary

Typhoid fever is endemic in Nepal, with Kathmandu coined “the typhoid capital of the world”. We developed a mathematical model to assess the importance of migration and antimicrobial resistance on the transmission of typhoid fever in Kathmandu, Nepal from April 1997 to June 2011. During this period, the burden of typhoid fever increased markedly from January 2000 to December 2003, after which the epidemic declined, but to a higher endemic level than in 2000. Our findings are consistent with the hypothesis that migration of susceptible individuals into Kathmandu played an important role in the epidemic, and may have been further aided by the emergence of typhoid fever with reduced susceptibility against fluoroquinolones. This study showed that identifying and targeting migrant populations with control efforts could be an important avenue to prevent typhoid transmission and disease.

## Introduction

Typhoid fever is a febrile disease, arising exclusively in humans, which is caused by the bacterium *Salmonella enterica* subspecies enterica serovar Typhi. *S*. Typhi is transmitted through contaminated food or water, and is a major cause of morbidity in countries plagued by lack of clean water or poor sanitation [1]. A substantial proportion of the typhoid fever burden occurs in South Asia, where the disease is widely endemic and is estimated to cause 3.7 to 7.0 million cases and 76,000 deaths annually [2–4].

Nepal is amongst the world’s most impoverished countries [5] and suffers from endemic typhoid fever [6,7]. Between 2000 and 2003, there was a marked increase in the number of *S*. Typhi cases at Patan Hospital, one of the main hospitals treating febrile patients in Kathmandu, rising from 245 cases in 2000 to 1,792 cases in 2003 [6]. The number of *S*. Typhi cases subsequently declined but to a higher endemic level than in 2000 [7]. This epidemic of *S*. Typhi coincided with the increased prevalence of organisms with reduced susceptibility against fluoroquinolones [8,9] and the emergence of *S*. Typhi H58 variants [10,11]. H58 *S*. Typhi are associated with reduced susceptibility against fluoroquinolones and other forms of antimicrobial resistance, and this genotype has become the dominant variant in many countries where *S*. Typhi is endemic [11,12]. Nepal additionally has a highly migratory population, which could also have fuelled the typhoid fever epidemics [13].

Mathematical modelling is a useful methodology for evaluating the hypotheses that underlie disease processes, which can be applied to assess the impact of control efforts and predict future trends. We have previously developed a mathematical model of typhoid fever transmission dynamics, which we used to predict the impact of vaccination on typhoid fever in South Asia [14], to explain the drivers of the emergence of typhoid fever in Malawi [15], and to determine the cost-effectiveness of vaccination strategies [16]. Here, we assessed the potential drivers associated with typhoid fever from April 1997 to June 2011 in Kathmandu, Nepal. We further adapted the previous model to investigate whether the epidemic of typhoid fever in Kathmandu was driven by heightened migration, the emergence of organisms with reduced susceptibility against fluoroquinolones and/or increased fitness, or a combination of these factors.

## Methods

### A model for typhoid fever transmission dynamics

We adapted our previous typhoid fever transmission model [14] to assess factors associated with *S*. Typhi transmission dynamics in Kathmandu, Nepal (S1 Fig). Briefly, we assumed that individuals in Kathmandu were born susceptible to *S*. Typhi infection (S_1_) and were infected at rate (λ_p_+ λ_w_), which is composed of short-cycle transmission from the immediate environment (λ_p_) and long-cycle transmission from the broader environment and contamination of water (λ_w_), the latter occurring with a slight delay. Infected participants with a primary infection (I_1_) were assumed to be infectious for a period of 1/δ and then either recovered (R), became chronic carriers (C) or succumbed to the infection. We assumed that a fraction (α) experienced disease-induced mortality, a fraction (θ) became chronic carriers, which varied with age [17], and that the remaining (1-α-θ) individuals recovered. Recovered individuals (R) did not have life-long protection; we assumed that immunity waned at rate ω, which resulted in individuals becoming partially susceptible (S_2_). Partially susceptible individuals could be re-infected, but we assumed that this infection was subclinical (I_2_) and thus not observed in our data. Participants that were infected sub-clinically either recovered (R) or became chronic carriers (C). Further, we assumed that all infected participants shed infectious particles into the broader environment (W) at rate γ and that the bacteria remained infectious for a period of 1/ζ. Chronic carriers and sub-clinically infected participants had a reduced infectiousness by factor r. Additionally, the model assumed that the environmental transmission parameter (β_w_) varied seasonally, e.g. due to seasonal variation in rainfall [18]. Model parameters are described in Table 1 and a more detailed description of the model, with model equations can be found in the S1 Text.

**Table 1:** Fixed model parameters.

Parameter definition	Symbol	Value	Source
Total population	N	100,000	Assumption^1^
Birth rate	B	30 live births per 1,000 per year	Census data
Natural mortality rate	μ	29.6 deaths per 1,000 per year	Census data
Mean duration of temporary immunity	1/ω	104 weeks	Assumption^2^[20]
Proportion of primary infections that die	α	0.001	Assumption [21]
Rate of shedding into the water supply	γ	1 infectious unit per week	Assumption^3^
Relative infectiousness of chronic and short-term carriers	r	0.05	Assumption [14,15]
Mean duration of infectiousness	1/δ	4 weeks	[22]
Fraction of infected who become chronic carriers	θ	0.003–0.011 depending on age	[17]
Rate of decay of infectious particles from water	ζ	1/3 weeks^-1^	[23]

^1^ Based on the approximate catchment area of Patan Hospital

^2^ Previous analyses have shown the model is not sensitive to this parameter [14]

^3^ This is not individually identifiable from β_w_

In preliminary analyses, we found that the basic reproductive number of short-cycle (R_0p_) and long-cycle (R_0w_) transmission were not well identified even though the overall basic reproductive number (R_0_ = R_0p_ + R_0w_, defined as the expected number of secondary cases from an infectious individual in a fully susceptible population) was well identified in the fitted models. Therefore, we attributed a fixed proportion of R_0_ to R_0p_ and R_0w_, and examined how the model fit and estimated parameters varied for different proportions. For our primary analysis, we assumed 20% of transmission occurred via the short-cycle and 80% via the long-cycle, as previous studies have found a large variety of genotypes within typhoid clusters in this setting, suggesting that most transmission occurred via the long-cycle [19]. Further, we observed that the model tended to over-predict the proportion of cases in those <5 years of age among participants in randomised clinical trials (RCTs) evaluating treatment strategies for typhoid fever. We assumed this was due to under-reporting of cases in this age group (e.g. due to different symptoms or less severe disease in children <5 years old and/or the exclusion of the <2 year olds from the RCTs) and allowed for a reduction in the reporting fraction (k) in this age group. We assessed the robustness of our results to these assumptions in sensitivity analyses.

### Model scenarios

We assessed five different scenarios that were all modifications of a baseline model, based on hypotheses regarding the possible drivers of typhoid transmission dynamics in Kathmandu. In the baseline model, we assumed no changes in the epidemiology and transmission of typhoid over time. We estimated the basic reproductive number (R_0_), the amplitude of seasonality (q) and seasonal offset (l) for the long-cycle transmission, and a reporting fraction (f) to scale the predicted number of infections to the number of cases observed (Table 2) (details given in S1 Text).

**Table 2:** Model parameter estimates and Akaike information criteria for best-fit models for each scenario.

Parameter definition	Symbol	Prior distribution	Baseline	Scenario 1	Scenario 2	Scenario 3	Scenario 4	Scenario 5
Basic reproductive number	R_0_	Uniform (0,10)	2.43	4.18	2.34	2.11	3.83	2.79
Amplitude of seasonal forcing (long-cycle transmission)	q	Uniform (0,1)	0.43	0.43	0.64	0.41	0.48	0.42
Seasonal offset parameter (timing of seasonal peak)	l	Uniform (0,50)	22.81	22.64	19.10	21.03	21.09	21.31
Reporting fraction	f	Uniform (0,1)	0.60	0.27	0.38	0.30	0.30	0.22
Reduction in reporting fraction for those aged 0–5 years	k	Uniform (0,1)	0.02	0.02	0.01	0.01	0.03	0.02
Beginning week of increase in duration of infectiousness or transmission rate	t_0_	Uniform (0,L)^1^	-	-	14 March 1999	2 January 2000	23 August 1998	13 September 1998
End week of increase in duration of infectiousness or transmission rate	t_1_	Uniform (0,L) ^1^	-	-	14 February 2002	3 June 2002	21 February 2005	22 August 2005
Magnitude of increase in duration of infectiousness or transmission rate	m	Uniform (0,10)	-	-	2.09	1.90	1.51	1.90
Beginning week of immigration	t_immig0_	Uniform (0,L) ^1^	-	8 January 2001	-	-	4 December 2000	14 May 2001
End week of immigration	t_immig1_	Uniform (0,L) ^1^	-	1 July 2002	-	-	18 March 2002	17 September 2001
Proportion of initial age-specific population that immigrates weekly^2^	immigra-tion/N_a_	Uniform (0,1)	-	0.029	-	-	0.025	0.089
Akaike information criteria			13,908	11,080	11,324	11,244	10,989	10,931

^1^L refers to the length of the time period (742 weeks); value was rounded to the nearest week

^2^ Individuals migrating were considered susceptible and were aged 15–25 yrs.

**Fig 1:**
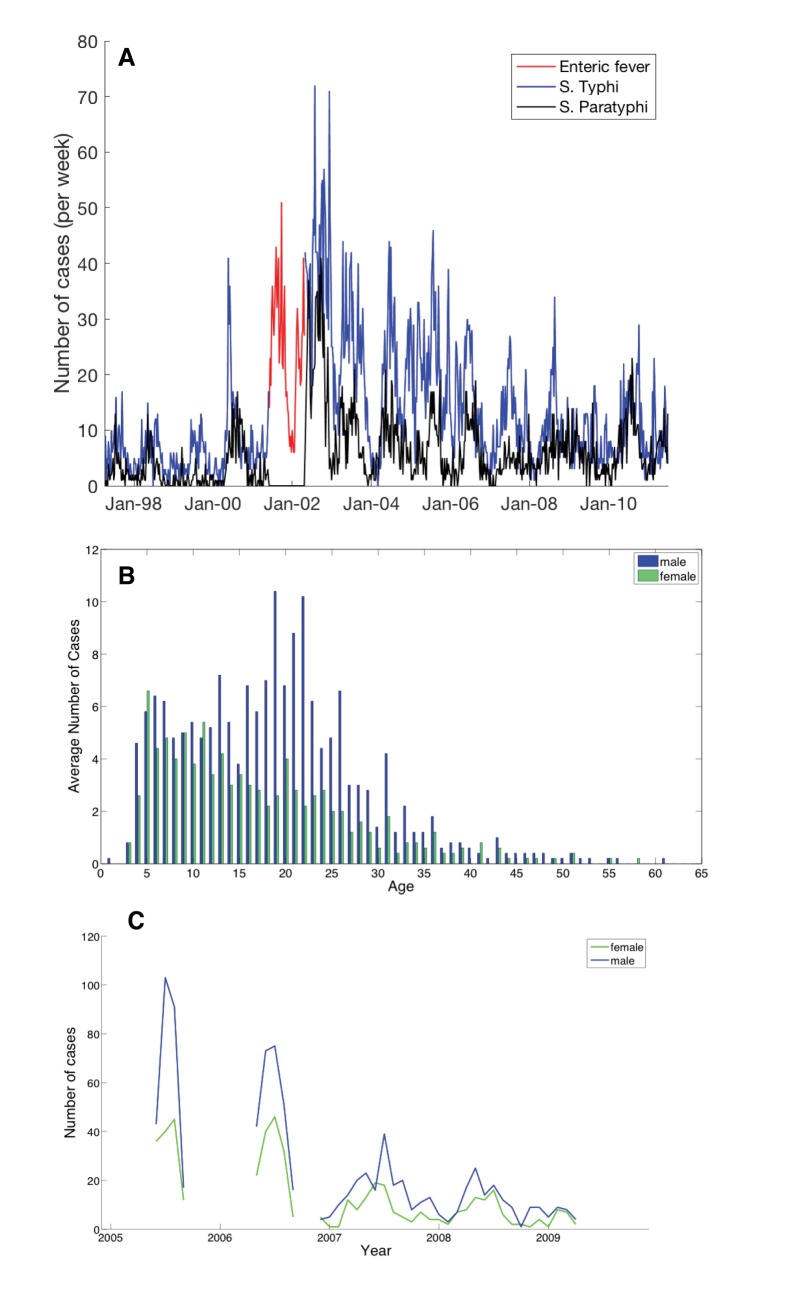
Overview of *Salmonella* Typhi, *S*. Paratyphi and enteric fever cases in Kathmandu, Nepal from April 1997 to June 2011. (A) Weekly number of *S*. Typhi cases (blue), *S*. Paratyphi cases (black) and enteric fever cases (red), from June 2001–May 2002; (B) the distribution of enteric fever cases by age during randomized controlled trials (RCTs) of typhoid treatment conducted between June 2005 and May 2009; (C) distribution of enteric fever cases by gender in the RCTs.

We observed a higher incidence of typhoid fever among men in young adulthood (Fig 1), particularly in 2005, and hypothesised that this was due to an influx of susceptible male workers that migrated to Kathmandu from rural settings where they were previously unexposed to typhoid (Scenario 1). We assumed that individuals in the 15–25 year old age groups migrated into the population and entered the fully susceptible state (*S*_1_) at a constant rate and estimated the week when immigration started (t_immig0_) and ended (t_immig1_, with t_immig1_>t_immig0_) and the number of weekly immigrants during that period (*immigration*), in addition to the four parameters in the baseline model (Table 2).

We also evaluated the emergence of antimicrobial resistant (AMR) *S*. Typhi, which in Nepal is primarily *S*. Typhi with reduced susceptibility against fluoroquinolones, on the transmission dynamics of typhoid by allowing for an increase in the duration of infectiousness (Scenario 2) or allowing for an increase in bacterial fitness, related to the emergence of H58 organisms (Scenario 3). For Scenario 2, we hypothesised that the emergence of AMR *S*. Typhi would increase the time taken to clear the infection, and therefore we allowed for a linear increase in the mean duration of infectiousness (1/δ). We estimated the time of emergence of AMR *S*. Typhi (t_0_), when the prevalence stabilized (t_1_, with t_1_>t_0_), the magnitude of the corresponding increase in the duration of infectiousness (m), as well as the parameters in the baseline model (Table 2). For Scenario 3, we assumed the increased fitness of AMR *S*. Typhi was associated with an improved growth rate [24], and therefore allowed for a linear increase in both the short- and long-cycle transmission parameters (β_p_ and β_w_). Similarly, we estimated the time of emergence (t_0_), when the prevalence stabilized (t_1_, with t_1_>t_0_), and the magnitude of the increase (m) in transmissibility, as well as the four parameters in the baseline model (Table 2).

Finally, we determined whether the pattern of typhoid fever in Kathmandu was the result of both the migration of susceptible individuals and the emergence of AMR *S*. Typhi organisms, either through an increase in duration of infectiousness (Scenario 4), for which we estimated all parameters in Scenarios 1 and 2, or an increase in bacterial fitness (Scenario 5), for which we similarly estimated all parameters in Scenarios 1 and 3 (Table 2).

For all scenarios, we conducted three sensitivity analyses. First, we allowed for a different appropriation of R_0_ to the long- and short-cycle pathways to assess the robustness of our results to the primary mode of disease transmission. Second, we assessed the sensitivity of the results to assumptions about the relative infectiousness of chronic carriers and individuals with subclinical infection, with two different fixed model parameters (details given in S1 Text). Finally, we determined whether the low proportion of cases in those aged 0–5 years was a result of reduced exposure, rather than reporting of cases, and allowed for a reduced short- and long-cycle force of infection for this age group.

### Data sources

Weekly data on the number of typhoid fever cases from April 1997 to June 2011 were obtained retrospectively from laboratory records at Patan hospital, Kathmandu. Patan hospital is a government hospital in the Kathmandu Valley, which provides emergency, out- and in-patient services. All inpatients and outpatients with suspected bacteraemia at Patan hospital have a blood culture performed. A case of enteric fever was defined as a positive blood culture for either *S*. Typhi or *Salmonella* Paratyphi A; the data consisted of laboratory records of the total number of blood culture confirmed enteric fever cases, as well as the number that were positive for *S*. Typhi or *S*. Paratyphi A. We did not have access to patient records. There were no known changes in diagnosis and blood culturing practices over the time period of the analysis. For one year, from June 2001 to May 2002, we only had data on the total number of enteric fever cases. Therefore, we estimated the missing number of *S*. Typhi and *S*. Paratyphi A cases for that year as the weekly number of enteric fever cases for that period times the proportion of enteric fever cases that were diagnosed as *S*. Typhi and *S*. Paratyphi A throughout the entire study period.

Sex- and age-specific data was obtained from patients in three RCTs conducted at Patan hospital evaluating the treatment of typhoid fever. The trials, all open-label randomised superiority trials, measured the treatment outcomes for gatifloxacin versus cefixime [25], gatifloxacin versus chloramphenicol [26], and gatifloxacin versus ofloxacin [27]. Eligible study participants were recruited between June 2005 and May 2009 from the emergency and outpatient department at Patan hospital. Participants had a fever for more than three days and were clinically diagnosed with enteric fever. Exclusion criteria applied to those who were pregnant, lactating, <2 years of age, weighing <10kg, had signs of severe typhoid fever (such as shock, jaundice or gastrointestinal bleeding), or had a previous known treatment with antimicrobials within a week of hospital admission (except when pretreated with amoxicillin or cotrimoxazole, as long as patients did not show evidence of clinical response). Additional details regarding the design and conduct of the RCTs can be found in Pandit et al. [25], Arjyal et al. [26] and Koirala et al. [27].

### Model fitting

We fit the model to weekly data on the number of *S*. Typhi cases between April 1997 and June 2011 and also to the age distribution of *S*. Typhi cases collected during the RCTs by maximum a posteriori estimation. To estimate the parameters for each model, we first specified an initial parameter set, within a reasonable parameter range. We then calculated the overall log-likelihood for each parameter set, which consisted of two components: (1) the log-likelihood of the number of weekly observed *S*. Typhi cases and (2) the log-likelihood of the age-specific number of *S*. Typhi cases (details given in S1 Text). Briefly, we assumed that the number of weekly observed *S*. Typhi cases was Poisson-distributed, with mean equal to the model-predicted number of cases (equal to the number of infections, summed over all age groups for each week, over the duration of infectiousness times the reporting fraction). For the log-likelihood of the age-specific number of *S*. Typhi cases we assumed that it was multinomially distributed, with the probability equal to the model-predicted proportion of cases in each age group (in five-year age groups from 0 to ≥80 years of age) and the number of events equal to the number of observed cases in each age group. We obtained an overall log-likelihood for each model by summing the log-likelihoods of the model fitted to the Poisson-distributed time series and multinomially distributed age distribution plus the prior log-likelihood of the model parameters. To determine the optimal parameter set, we minimised the negative overall log-likelihood using a simplex search method (using the ‘fminsearch’ command in MATLAB 8.6.0) and the parameter set with the highest posterior probability was selected to obtain the best-fit model for each scenario. We assumed uniform prior distributions for all model parameters and calculated the Akaike information criteria (AIC) for model comparison for each best-fit model (Table 2). Moreover, we assessed whether our parameter estimates converged by varying the initial starting values ten times.

## Results

**Fig 2:**
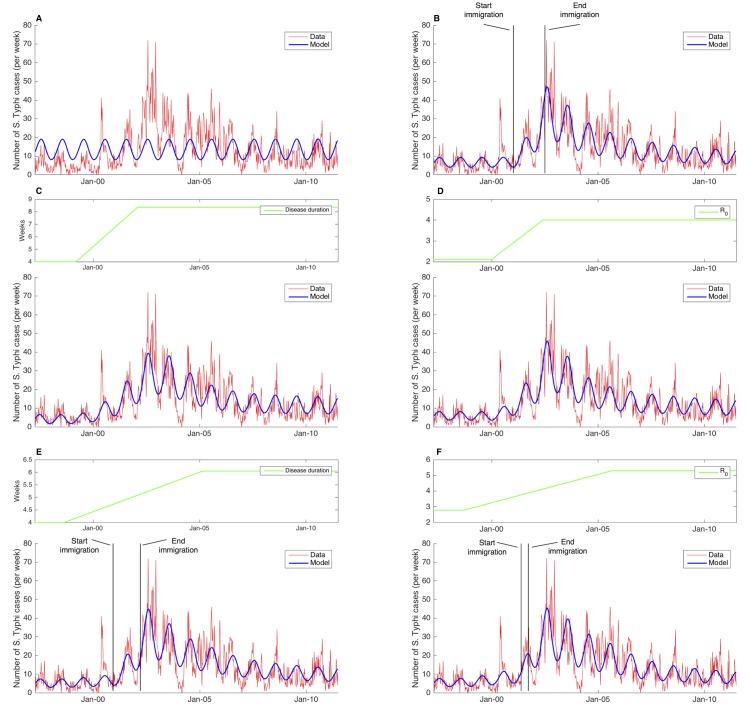
Fit of models for the baseline scenario and scenarios 1–5 to weekly number of *Salmonella* Typhi cases, from April 1997 to June 2011, in Kathmandu Nepal. (A) Baseline scenario; (B) Scenario 1; (C) Scenario 2; (D) Scenario 3; (E) Scenario 4; (F) Scenario 5. Observed weekly cases of *S*. Typhi (red); best-fit model (blue); Disease duration or R_0_ (green), depending on the scenario; vertical lines (black) indicate duration of migration.

The best-fit baseline model did not adequately predict long-term trends in *S*. Typhi cases (Fig 2A). This model overestimated the number of cases from 1997 to 2000 and failed to capture the increase in cases between 2000 and 2007. For scenario 1, which assumed a constant weekly migration of fully susceptible individuals 15–25 years of age, the best-fit model provided a better fit to the data and accurately predicted the trend of cases (Fig 2B). Notably, the best-fit model identified a peak of 47 cases in August 2002, which was consistent with the peak in the real data. In this scenario, the model estimated that 342 susceptible people migrated weekly between January 2001 and July 2002. Although the best-fit model captured nearly all the seasonal peaks of *S*. Typhi, it failed to predict the peak in May 2000.

Scenario 2 assumed that the emergence of AMR *S*. Typhi would lead to an increase in the duration of infectiousness. The best-fit model again provided a reasonably good fit to the data, although with a higher AIC compared to scenario 1 (Fig 2C). The best-fit model appeared to capture all peaks in the observed data and estimated an increase in the duration of infectiousness from 4 to 8.4 weeks between March 1999 and February 2002.

When we assumed that the increase in *S*. Typhi was a consequence of elevated bacterial fitness and thus an increase in the transmission parameters (scenario 3), the best-fit model again showed an acceptable fit to the observed cases similar to scenario 2 and with a higher AIC than scenario 1 (Fig 2D). The basic reproductive number in this scenario increased from 2.1 to 4.0 between January 2000 and June 2002.

Scenario 4 assumed a constant weekly migration of 15–25 year old susceptible individuals and the emergence of AMR *S*. Typhi throughout the study period, resulting in an increase in the duration of infectiousness. The best-fit model estimated a weekly migration of 296 susceptible people between December 2000 and March 2002 and an increase in the duration of infectiousness from 4.0 to 6.0 weeks between August 1998 and February 2005 (Fig 2E). The duration of migration was slightly shorter than in scenario 1, although the estimated rate of migration was comparable. Additionally, the increase in duration of infectiousness was protracted in this scenario in comparison to scenario 2 and plateaued at a shorter duration of infectiousness than in scenario 2. The best-fit model provided a good fit to the data and performed better, with respect to AIC, than the best-fit model in scenario 2 and comparably to the model in scenario 1.

Scenario 5 mimicked scenario 4, although in this case the emergence of *S*. Typhi was attributed to an increase in bacterial fitness. The best-fit model predicted a weekly migration of 1043 susceptible people from May to September 2001 (Fig 2F). This was a shorter time period than in scenario 1, although the rate of people migrating was more than twice as large as in scenario 1. The model also estimated an increase in the transmissibility of *S*. Typhi, with R_0_ increasing from 2.8 to 5.3 between September 1998 and August 2005. In terms of AIC, the model performed equally as well as the best-fit models in scenarios 1 and 4.

**Fig 3:**
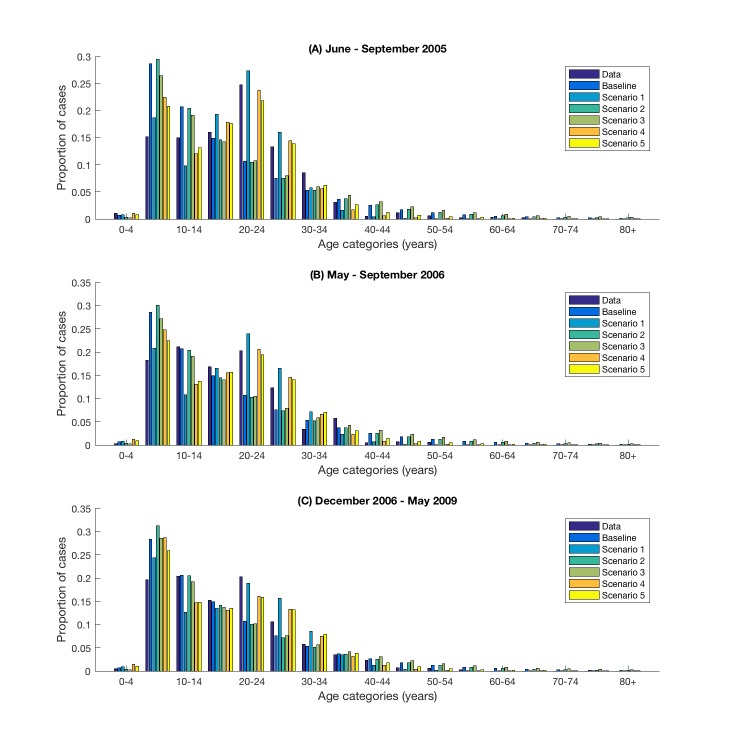
Observed and model-predicted age distribution of *Salmonella* Typhi cases. Observed and model-predicted age distribution shown for the three time periods for which age-specific data on cases was available. Age-specific data of *S*. Typhi cases from (A) June to September 2005, (B) from May to September 2006, and (C) from December 2006 to May 2009.

All models fit the age distribution of *S*. Typhi cases among children under the age of five years, adolescents aged 15 to 19 and those older than 30 years reasonably well (Fig 3). However, the model-predicted age distribution for cases in the other age categories varied widely between scenarios. In particular, scenarios 2 and 3, which provided a poorer fit to the weekly number of cases, underestimated the number of typhoid cases in the 20–29 year olds. However, the latter two scenarios provided the best fit for the proportion of cases aged 10 to 14 years, while scenarios 1, 4 and 5 under-predicted this proportion of cases, particularly in 2005. All models over-predicted the proportion of cases among 5–9 year-olds.

**Fig 4:**
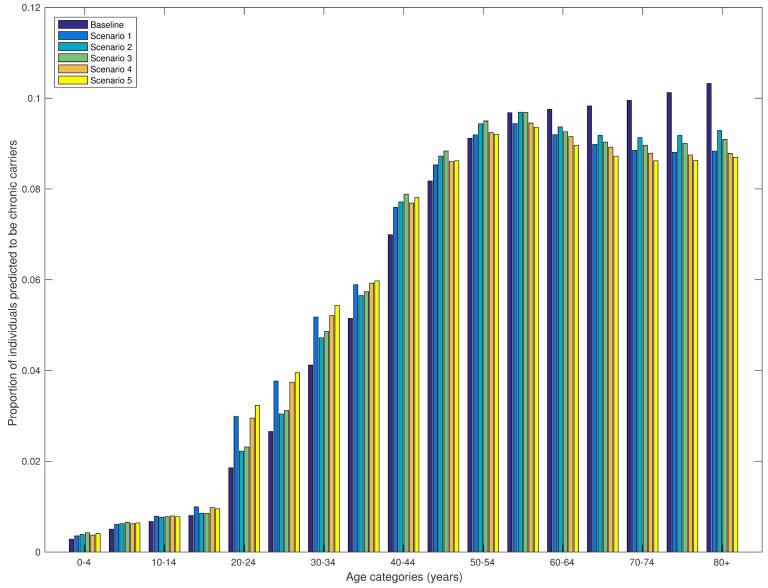
Model-predicted proportion of chronic carriers by age categories for different model scenarios. The proportion of individuals in each age group predicted to be in the chronic carrier state is plotted for each model scenario.

All model scenarios predicted a similar proportion of chronic carriers for each age category (Fig 4). The proportion of chronic carriers among those younger than 20 years was up to 1%, after which the proportion increased linearly until it reached a maximum of approximately 10% for those 50 years or older.

Our models converged to comparable parameter estimates, and the findings were generally robust to attributing different proportions of R_0_ to R_0p_ and R_0w_ except when R_0_ was wholly attributed to the short-cycle basic reproductive number (S2 and S3 Figs). Moreover, reducing or increasing the relative infectiousness of chronic and short-term carriers in the sensitivity analysis resulted in lower and higher values of R_0,_ respectively, but the best-fit models still provided a good fit to the data (S4 and S5 Figs). Finally, attributing the low proportion of *S*. Typhi cases in those under five years of age to reduced short- and long-cycle exposure resulted in best-fit models that did not differ, in terms of AIC, from the best-fit models in the main analysis, with the models again providing a good fit to the weekly number of *S*. Typhi cases and to the age distribution of cases (S6 and S7 Figs).

## Discussion

Typhoid fever is endemic in Nepal, with Kathmandu coined “the typhoid capital of the world” [28]. We assessed the drivers of typhoid fever in Kathmandu from April 1997 to June 2011. During this period, the burden of typhoid fever increased markedly from January 2000 to December 2003, after which the epidemic declined, but to a higher endemic level than in 2000. Our analysis is consistent with the hypothesis that the epidemics were caused by the migration of susceptible individuals to Kathmandu and aided by the emergence of *S*. Typhi with reduced susceptibility against fluoroquinolones.

The burden of typhoid fever in rural Nepal has been recently estimated to be commonly over diagnosed, as the prevalence of blood culture confirmed cases was low (<1%), suggesting that individuals in rural settings have low immunity to infection [29]. In Nepal, young men often migrate from rural areas to cities in their mid- to late-teens to pursue further education or in search of employment. A survey by the Nepalese government in 2007 suggested that high rates of internal migration occur. They identified that in nearly 40% of the country’s households, at least one person travelled away from home for employment in the last 12 months, with those most likely to migrate in search of employment being men between 15 and 30 years of age [13]. The timing of the migration estimated by our best-fit models, from December 2000 to July 2002, also coincided with a period of political instability in Nepal. During this period, Nepal experienced an increase in violence due to Maoist rebel forces, especially in the Nepalese countryside, which also was likely to impact dramatically on migration from rural areas to Kathmandu [30].

The emergence of AMR *S*. Typhi organisms is a well-established problem in South and Southeast Asia. Following the emergence of multi-drug resistant (MDR) *S*. Typhi, which are resistant to chloramphenicol, amoxicillin and co-trimoxazole, in the 1990s, fluoroquinolones have been the preferred treatment option [31]. However, the widespread use of fluoroquinolones in Nepal in since the mid 1990s has led to a shift in the drug resistance pattern; an increase in *S*. Typhi exhibiting reduced susceptibility to fluoroquinolones (as indicated by resistance to nalidixic acid) coincided with a decline in the prevalence of MDR *S*. Typhi [8,9]. Moreover, in Nepal, the increase in typhoid fever cases broadly corresponds to the introduction of the dominant H58 lineage [10–12]. The H58 *S*. Typhi variants have been associated with reduced susceptibility to fluoroquinolones, particularly in South and Southeast Asia [11]. The reduced susceptibility to fluoroquinolones is due to mutations in the DNA gyrase gene (*gyrA*) and the topoisomerase gene (*parC*) [32,33]. These mutations are not associated with a fitness cost, and may even impart a fitness advantage, enabling widespread proliferation in the absence of antimicrobial pressure [24].

The emergence of *S*. Typhi with reduced susceptibility to fluoroquinolones and/or introduction of H58 variants were supported by our best-fit models, which predicted an increase in disease duration or transmissibility beginning in August/September 1998. While this is broadly consistent with the timing of the emergence of the H58 haplotype and reduced susceptibility to fluoroquinolones in Nepal [9,11], the scenarios we modelled were agnostic to the specific AMR profile. A comparable analysis of the recent emergence of typhoid fever in Blantyre, Malawi, identified MDR *S*. Typhi and/or the emergence of H58 variants as the primary driver of the increased number of typhoid fever cases [15]. However, typhoid fever was previously endemic in Kathmandu, and therefore our model suggests the impact of the emergence of H58 and associated AMR would not have been as profound as in Blantyre, where previously a very low incidence of *S*. Typhi infections was reported [15,34,35].

The sex- and age-specific data that led to the investigation of migration as a potential driver of the increase in typhoid cases in Kathmandu and to which the models were fitted were from participants enrolled in clinical trials. These age-specific data also showed a very low number of *S*. Typhi cases among those aged under five years. This was mostly likely due to the exclusion of <2 years olds from the RCTs; we accounted for this by allowing the reporting rate of cases to vary in the <5 year olds. In contrast, in Dehli (India) and Dhaka (Bangladesh), <5 year olds were amongst those with the highest number of typhoid fever cases [36,37]. However, it is also possible that the dearth of cases in this age group is due to reduced exposure of young children to *S*. Typhi or different or less severe symptoms in young children. Understanding the true age distribution of typhoid fever burden, particularly in young children, has important implications when considering typhoid vaccination strategies in this setting.

Chronic carriage is an important but poorly understood part of *S*. Typhi epidemiology. A proportion of infected cases will become chronic carriers, defined as shedding typhoid in their stool for at least one year; the vast majority of these individuals are asymptomatic [38]. Chronic carriage has been associated with gallbladder disease, and previous studies have found that *S*. Typhi was prevalent in 3% of individuals in Kathmandu, aged 23 to 39 years, undergoing cholecystectomy [39]. This is congruent with the model-predicted proportion of chronic carriers among those aged 20 to 40 years, which was predicted to be 2–6% in these age groups (Fig 4).

Other *Salmonella* serovars are also prevalent in South and Southeast Asia, such as *S*. Paratyphi A in Nepal [7] and non-typhoidal *Salmonella* (NTS) in Vietnam [40], and may confer cross-protective immunity to *S*. Typhi infection. This hypothesis was evaluated for NTS in Malawi using a comparable mathematical modelling approach, but was not found to explain the pattern of *S*. Typhi infections [15]. The natural history of *S*. Paratyphi A is not fully understood, which would complicate the incorporation of *S*. Paratyphi A in our model. However, the interaction amongst *Salmonella* serovars remains a subject that should be further explored.

An important caveat of our approach is that our model is an over simplification of typhoid fever transmission dynamics. We assumed homogeneous mixing, such that susceptible and infected individuals randomly come in contact. Moreover, we used age- and sex-specific data from RCTs between 2005 and 2009, which does not overlap with the estimated time period of migration in our best-fit models. Unfortunately, no other age- or sex-specific data on the patient population or data on migratory patterns in Nepal was available. Furthermore, we did not account for spatial patterns in the distribution of cases nor for potential environmental and host risk factors (aside from age) [18,19]. Many of these factors are the subject of on-going research [41–43], and evidence from epidemiological and clinical studies concerning these factors will enable us to incorporate this in future mathematical models.

Our findings are consistent with the hypothesis that the increase in *S*. Typhi infections in Kathmandu, Nepal, was due to the migration of susceptible male workers into Kathmandu and may have been further aided by an increase in *S*. Typhi with reduced susceptibility to fluoroquinolones. The emergence of AMR typhoid fever is an important public health problem in Nepal, with few antimicrobial drugs remaining as treatment options [44,45]. This underlines the importance of vaccination and other control measures, such as water, sanitation and hygiene approaches, to prevent disease transmission and infection. Identifying and targeting migrant populations should be an important component of these efforts.

## Supporting information

S1 Text Detailed methods and sensitivity analyses. (DOCX)

S1 Fig Diagram of model structure. (TIFF)

S2 Fig Fit of models for scenarios 1–5 to weekly number of *Salmonella* Typhi cases, from April 1997 to June 2011, in Kathmandu Nepal, with different proportions of R_0_ to R_0p_ and R_0w_. (A) Scenario 1; (B) Scenario 2; (C) Scenario 3; (D) Scenario 4; (E) Scenario 5. Observed weekly cases of S. Typhi (red); best-fit model with 0% R_0p_ and 100% R_0w_ transmission (navy); best-fit model with 20% R_0p_ and 80% R_0w_ transmission (magenta); best-fit model with 40% R_0p_ and 60% R_0w_ transmission (yellow); best-fit model with 60% R_0p_ and 40% R_0w_ transmission (purple); best-fit model with 80% R_0p_ and 20% R_0w_ transmission (green); best-fit model with 100% R_0p_ and 0% R_0w_ transmission (light-blue).
(TIFF)

S3 Fig Observed and model-predicted age distribution, for different proportions of R_0_ to R_0p_ and R_0w,_ of *Salmonella* Typhi. Observed and model-predicted age distribution shown for the three time periods for which age-specific data on cases was available. Columns represent the age distribution for a given time period while the rows indicate the age distribution for a given model scenario.
(TIFF)

S4 Fig Fit of models for scenarios 1–5 to weekly number of *Salmonella* Typhi cases, from April 1997 to June 2011, in Kathmandu Nepal, with different value for relative infectiousness of chronic carriers and subclinical infection (r). (A) Scenario 1; (B) Scenario 2; (C) Scenario 3; (D) Scenario 4; (E) Scenario 5. Observed weekly cases of S. Typhi (red); best-fit model with r = 0.01 (blue); best-fit model with r = 0.05 (green); best-fit model with r = 0.25 (magenta).
(TIFF)

S5 Fig Observed and model-predicted age distribution, for different values of relative infectiousness of chronic carriers and subclinical infection (r), of *Salmonella* Typhi. Observed and model-predicted age distribution shown for the three time periods for which age-specific data on cases was available. Columns represent the age distribution for a given time period while the rows indicate the age distribution for a given model scenario.
(TIFF)

S6 Fig Fit of models for scenarios 1–5 to weekly number of *Salmonella* Typhi cases, from April 1997 to June 2011, in Kathmandu Nepal, with the low proportion of cases in those aged 0–5 years attributed to (1) the under-reporting of cases or (2) a reduced exposure in this age group. (A) Scenario 1; (B) Scenario 2; (C) Scenario 3; (D) Scenario 4; (E) Scenario 5. Observed weekly cases of S. Typhi (red); best-fit model with under-reporting (blue); best-fit model with reduced exposure (green).
(TIFF)

S7 Fig Observed and model-predicted age distribution, attributing the low proportion of cases in those aged <5 years due to under-reporting of cases or a reduced exposure in this age group, of *Salmonella* Typhi. Observed and model-predicted age distribution shown for the three time periods for which age-specific data on cases was available. Columns represent the age distribution for a given time period while the rows indicate the age distribution for a given model scenario.
(TIFF)

S1 Data Weekly data of *Salmonella* Typhi cases between April 1997 and June 2011. (CSV)

S2 Data Age distribution of *Salmonella* Typhi cases collected during the randomized controlled trials. (CSV)
