# Supplementary material for: The impact of migration and antimicrobial resistance on the transmission dynamics of typhoid fever in Kathmandu, Nepal: A mathematical modelling study
Source: PLoS Negl Trop Dis. 2017 May 5;11(5):e0005547. doi: 10.1371/journal.pntd.0005547 (PMC5435358; doi:10.1371/journal.pntd.0005547)
Supplement: S1 Text — (DOCX) [file pntd.0005547.s001.docx]

# **SUPPORTING INFORMATION**

# **The impact of migration and antimicrobial resistance on the transmission dynamics of typhoid fever in Kathmandu, Nepal: a mathematical modelling study**

Neil J. Saad^1*^, Cayley C. Bowles^2^, Bryan T. Grenfell^3,4^, Buddha Basnyat^5,6^, Amit, Arjyal^5^, Sabina Dongol^5^, Abhilasha Karkey^5^, Stephen Baker^6,7^ and Virginia E. Pitzer^1,4^

^1^Department of Epidemiology of Microbial Disease, Yale School of Public Health, Yale University, New Haven, Connecticut, United States of America;

^2^David Geffen School of Medicine, University of California Los Angeles, Los Angeles, California, United States of America;

^3^Department of Ecology and Evolutionary Biology, Princeton University, Princeton, New Jersey, United States of America;

^4^Fogarty International Center, Bethesda, Maryland, United States of America;

^5^Oxford University Clinical Research Unit, Patan Academy of Health Sciences, Kathmandu, Nepal;

^6^Centre for Tropical Medicine and Global Health, Oxford University, United Kingdom;

^7^The Hospital for Tropical Diseases, Wellcome Trust Major Overseas Programme, Oxford University Clinical Research Unit, Ho Chi Minh City, Vietnam;

Corresponding author: neil.saad@yale.edu

**Text S1 Contents**

1. Methods

1.1. Typhoid fever transmission model structure and equations

1.2. Model fitting

1.3. Model scenarios

2. Sensitivity analyses

2.1. Basic reproductive number of short- and long-cycle transmission

2.2. Chronic carriage and relative infectiousness

2.3. Over prediction of cases among those under five years

# Methods

## Typhoid fever transmission model structure and equations

We adapted our previous model [1] to assess the hypotheses of *Salmonella* Typhi transmission dynamics in Kathmandu, Nepal and show the model structure in Figure S1. We incorporated age structure into the model by employing discrete age categories (in five-year age groups from 0 to ≥80 years of age), with the assumption of exponential aging from one category to the next. Therefore, each model compartment, with the exception of W, is composed of a set of age-specific compartments, e.g. $\boldsymbol{S}_{\boldsymbol{1}}\boldsymbol{=\{}S_{1,1},S_{1,2}, \ldots, {S_{1,a}, \ldots,S}_{1,17}\boldsymbol{\}}$, with *a* indicating the age-specific compartment. Further, we assumed homogeneous mixing and frequency-dependent long- and short-cycle transmission.

The equations for the age-structured model are as follows:

$$\frac{{dS}_{1,a}}{dt}=B_{a}-\left( \lambda_{p,a}+\lambda_{w} \right)S_{1,a}- {\mu S}_{1,a}$$

$$\frac{{dI}_{1,a}}{dt}=\left( \lambda_{p,a}+\lambda_{w} \right)S_{1,a}- \delta I_{1,a}- {\mu I}_{1,a}$$

$$\frac{dR_{a}}{dt}=\delta\left( 1-\alpha-\theta_{a} \right)I_{1,a}+\delta(1-\theta_{a})I_{2,a}- \omega R_{a}- \mu R_{a}$$

$$\frac{dC_{a}}{dt}=\delta\theta_{a}(I_{1,a}+I_{2,a})- \mu C_{a}$$

$$\frac{{dS}_{2,a}}{dt}=\omega R_{a}-\left( \lambda_{p,a}+\lambda_{w} \right)S_{2,a}- {\mu S}_{2,a}$$

$$\frac{{dI}_{2,a}}{dt}=\left( \lambda_{p,a}+\lambda_{w} \right)S_{2,a}- \delta I_{2,a}- {\mu I}_{2,a}$$

$$\frac{dW}{dt}=\gamma\left( I_{1,a}+{rI}_{2,a}+rC_{a} \right)- \xi W$$

with *a* indicating the age-specific compartment and parameters:

B*_a_*, the number of new births at time *t* (which is only ≠ 0 if a=1, i.e. the youngest age group, and 0 otherwise); λ_p,a_, the short-cycle force of infection; λ_w_, the long-cycle force of infection; μ, the natural mortality rate; δ, the rate of recovery from infectiousness; α, the disease-induced mortality rate; θ_a_, the fraction of infectious individuals who go on to become chronic carriers (which varies by age); ω, the rate of waning clinical immunity; γ, the rate of shedding into the water supply (W); r, relative infectiousness of chronic carriers (C) and subclinical infections (I_2_); ξ, rate of decay of infectious particles from the water supply.

We assumed that long-cycle transmission would be affected by rainfall and assumed it varied according to a sinusoidal seasonal forcing function. The short-cycle (λ_p,a_) and long-cycle (λ_w_) force of infection are given by:

$$\lambda_{p,a}= \frac{\beta_{p}(I_{1,a}+ rI_{2,a}+rC_{a})}{N}$$

$$\lambda_{w}= \frac{\beta_{w}\left( 1+q\cos\left( \frac{2\pi t-l}{52.18} \right) \right)W}{N}$$

with *a* indicating the age-specific compartment and parameters:

$\beta_{p}$, the short-cycle transmission rate; $\beta_{w}$, the long-cycle transmission rate; q, the amplitude of seasonal forcing: l, the seasonal offset parameter; N, the total population size. We estimated q and l using maximum a posteriori estimation (see section on model fitting). We derived $\beta_{p}$ and $\beta_{w}$ from the short- and long-cycle basic reproductive numbers, $R_{0p}$ and $R_{0w}$ respectively, as follows:

$$\beta_{p}= \frac{R_{0p}(\mu+\delta)}{(1+\left( \frac{r\delta}{\mu}\frac{\sum_{a=1}^{17} {N_{a}\theta}_{a}}{\sum_{a=1}^{17} N_{a}} \right))}$$

$$\beta_{w}= \frac{R_{0w}\xi(\mu+\delta)}{\gamma(1+\left( \frac{r\delta}{\mu}\frac{\sum_{a=1}^{17} {N_{a}\theta}_{a}}{\sum_{a=1}^{17} N_{a}} \right)))}$$

The model was simulated for a “burn-in” period of 50 years until a quasi-equilibrium was reached. We verified that the equilibrium population age distribution was similar to that of Kathmandu in 2000.

## Model scenarios

We assessed five different scenarios that were all modifications of the baseline model described in Section 1.1. For scenario 1 (and 4 and 5), we assumed that individuals in the 15-25 year old age groups migrated into the population and entered the fully susceptible state (S_1_) at a constant rate. This resulted in the following model equation for the fully susceptible state (S_1_):

$$\frac{{dS}_{1,a}}{dt}=B_{a}+{immigration}_{a}-\left( \lambda_{p,a}+\lambda_{w} \right)S_{1,a}- {\mu S}_{1,a}$$

with *immigration_a_* equal to the age-specific number of susceptible individuals that migrated into Kathmandu each week between the time when the immigration started (t_immig0_) and ended (t_immig1_, with t_immig1_>t_immig0_), and the other parameters as described above in Section 1.1.

For scenario 2 (and 4), we hypothesised that the emergence of antimicrobial resistant (AMR) *S.* Typhi would increase the time taken to clear the infection. Therefore, we allowed the mean duration of infectiousness to increase linearly between time t_0_ and time t_1_ (with t_1_>t_0_), after which the duration of infectiousness would remain constant, with the duration then equalling *m* (the magnitude of increase in duration) times the original duration of infectiousness; hence, the rate of recovery from infectiousness decreased from *δ* to *δ*/*m*.

Similarly, for scenario 3 (and 5), we assumed the increased fitness of AMR S. Typhi was associated with an improved growth rate. In this case we allowed for short- and long-cycle transmission parameters (β_p_ and β_w_) to increase linearly between time t_0_ and time t_1_ (with t_1_>t_0_), after which β_p_ and β_w_ would remain constant and equal to *m*β_p_ and *m*β_w_, respectively.

## Model fitting

We fit the model to weekly data on *S.* Typhi cases between April 1997 to June 2011 and to the age distribution of *S.* Typhi cases collected during the treatment trials by maximum a posteriori estimation. We calculated the log-likelihood of the data, which consisted of two components: (1) the log-likelihood of the number of weekly observed *S.* Typhi cases and (2) the log-likelihood of the age-specific number of *S.* Typhi cases.

We assumed that the log-likelihood of the number weekly observed *S.* Typhi cases was Poisson-distributed with a mean equal to the model-predicted number of cases:

$${LL}_{time series}= \sum_{w} \sum_{a} C_{w,a}\log\left( D_{w,a} \right)- D_{w,a}- \sum_{i=1}^{C_{w,a}} log(i)$$

With $C_{w,a}$, the number of observed cases in week w and age group a; and $D_{w,a}$ , the number of model-predicted clinical infections over the duration of infectiousness times the reporting fraction (i.e. $D_{w,a}=f\delta I_{1,w,a}$).

We assumed that the log-likelihood of the age-specific number of *S.* Typhi cases was multinomially distributed, with the probability equal to the model-predicted proportion of cases and the number of events equal to the number of observed cases:

$${LL}_{age distribution}= \sum_{a} x_{a}\log\left( P_{a} \right)$$

with $x_{a}$, the number of observed cases in age group a; and $P_{a}$ the model-predicted proportion of observed cases in age group a. Data on the age-specific number of *S.* Typhi cases was available for three separate time periods (June to September 2005; May to September 2006; December 2006 to May 2009) and we therefore calculated the log-likelihood for each of these time periods.

The overall log-likelihood was the sum of the log-likelihood components plus the prior log-likelihood of the model parameters:

$${LL}_{Total}={LL}_{time series}+{LL}_{age distribution 2005}+{LL}_{age distribution 2006}+{LL}_{age distribution 2006-2009}+{LL}_{prior}$$

To estimate the parameters for each model, we first specified an initial parameter set, within a reasonable parameter range. We then calculated the overall log-likelihood for each parameter set. Finally, we minimised the negative overall log-likelihood using a simplex search method (using the ‘fminsearch’ command in MATLAB 8.6.0) and the parameter set with the highest posterior probability was selected to obtain the best-fit model for each scenario.

# Sensitivity analyses

## Basic reproductive number of short- and long-cycle transmission

We attributed a fixed proportion of R_0_ to R_0p_ and R_0w_ in the model because we found that the basic reproductive number of short-cycle (R_0p_) and long-cycle (R_0w_) transmission were not well identified even though the overall basic reproductive number (R_0_ = R_0p_ + R_0w_) was well identified in the fitted models. In our main analysis, we assumed 20% of transmission occurred via the short-cycle and 80% via the long-cycle based on genotyping data indicating that even within the same household, individuals are often infected with different strains of *S.* Typhi [2]. We examined how the model fit and estimated parameters varied for different proportions. Figure S2 shows that our results findings were generally robust to attributing different proportions of R_0_ to R_0p_ and R_0w_ except when R_0_ was wholly attributed to the R_0p_. In the latter case, the best-fit model failed to capture the seasonality of *S.* Typhi and provided a poor fit to the observed weekly cases. Figure S3 shows the age distribution of *S.* Typhi cases for three different time periods and the best-fit models do not appear to be influenced by allocating different proportions of R_0_ to R_0p_ and R_0w_.

## Chronic carriage and relative infectiousness

The role of chronic carriage is important but poorly understood. In the main analysis we assumed that individuals with chronic carriage and subclinical infection had a reduced infectiousness (r) of 5%, based on previous estimates from typhoid fever transmission models [1,3]. We assessed the sensitivity of our results to this assumption by varying the parameter with two other estimates, 1% and 25%, which were previous extreme lower and upper estimates, respectively, from typhoid fever transmission models [1,3]. The best-fit models provided a comparable fit to the observed weekly *S.* Typhi cases and the age distribution of *S.* Typhi cases (Figure S4 and S5). Reducing or increasing the relative infectiousness resulted in lower and higher values of R_0_, respectively, although the best-fit models with a relative infectiousness of 5% provided the lowest AIC compared to the best-fit models with 1% and 25%.

## Over-prediction of cases among those under five years

In preliminary analyses we also observed that the model tended to over predict the proportion of cases in those <5 years of age. For the primary analysis we assumed this was due to under-reporting of cases in this age group and allowed for a reduction in the reporting fraction in this age group by a factor k. However, in a sensitivity analysis we assessed whether the low proportion of cases in those aged <5 years was a result of reduced exposure, rather than reporting of cases, and allowed for a reduced short- and long-cycle force of infection for this age group by allowing for a reduction in the force of infection by a factor k. We found that the best-fit models in this sensitivity analysis did not differ, in terms of AIC, from the best-fit models in the main analysis, with the models providing a good fit the weekly number of *S.* Typhi cases and to the age distribution of the cases (Figures S6 and S7).

**References**

1. Pitzer VE, Bowles CC, Baker S, Kang G, Balaji V, Farrar JJ, et al. Predicting the Impact of Vaccination on the Transmission Dynamics of Typhoid in South Asia: A Mathematical Modeling Study. PLoS Negl Trop Dis. 2014;8: 40. doi:10.1371/journal.pntd.0002642

2. Baker S, Holt KE, Clements ACA, Karkey A, Arjyal A, Boni MF, et al. Combined high-resolution genotyping and geospatial analysis reveals modes of endemic urban typhoid fever transmission. Open Biol. 2011;1: 110008. doi:10.1098/rsob.110008

3. Pitzer VE, Feasey NA, Msefula C, Mallewa J, Kennedy N, Dube Q, et al. Mathematical modeling to assess the drivers of the recent emergence of typhoid fever in Blantyre, Malawi. Clin Infect Dis. 2015;61: S251–S258. doi:10.1093/cid/civ710
