# Supplementary figures and images for: The impact of migration and antimicrobial resistance on the transmission dynamics of typhoid fever in Kathmandu, Nepal: A mathematical modelling study
Source: PLoS Negl Trop Dis. 2017 May 5;11(5):e0005547. doi: 10.1371/journal.pntd.0005547 (PMC5435358; doi:10.1371/journal.pntd.0005547)

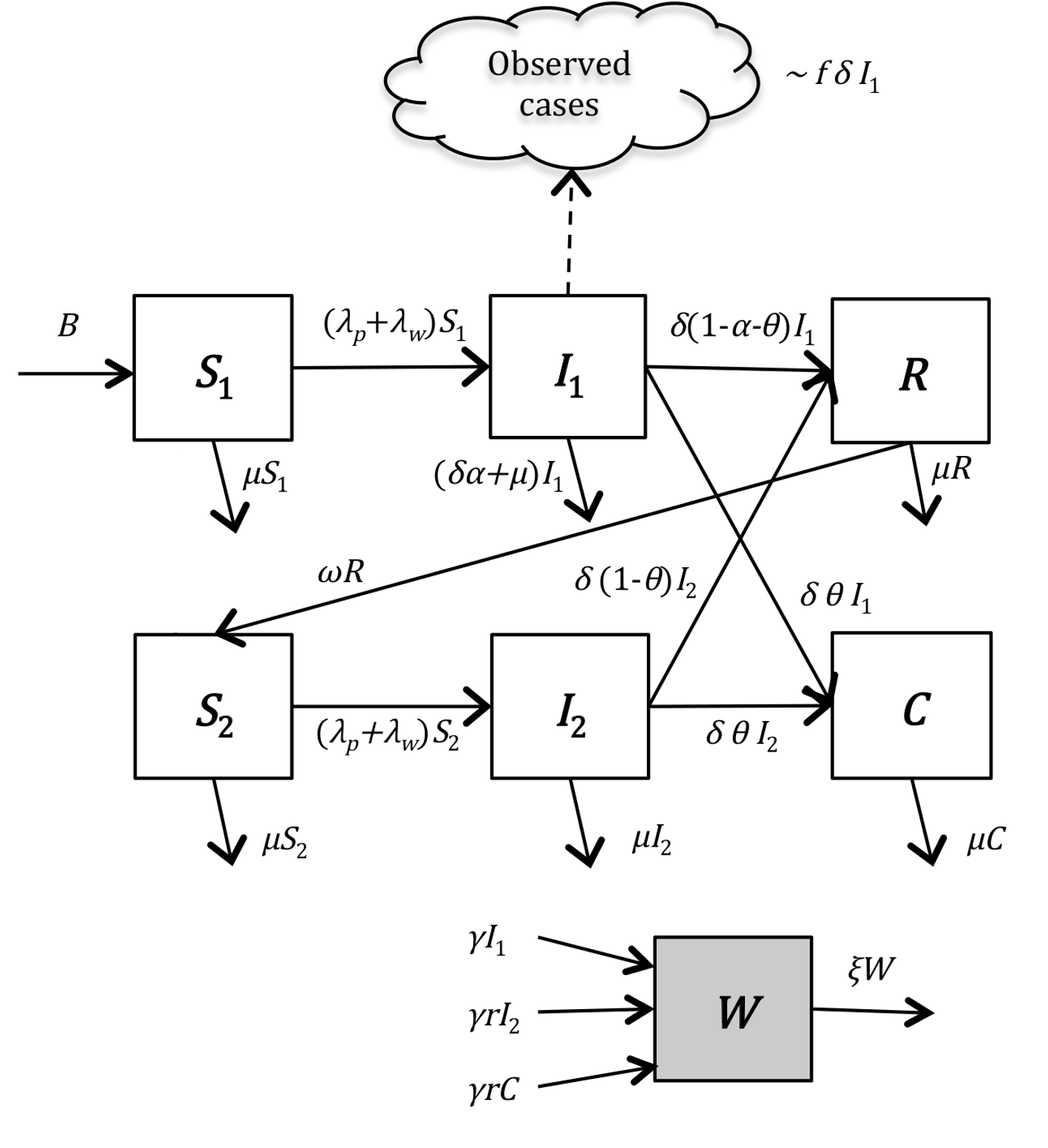

Supplement: S1 Fig — (TIFF) [file pntd.0005547.s002.tiff]

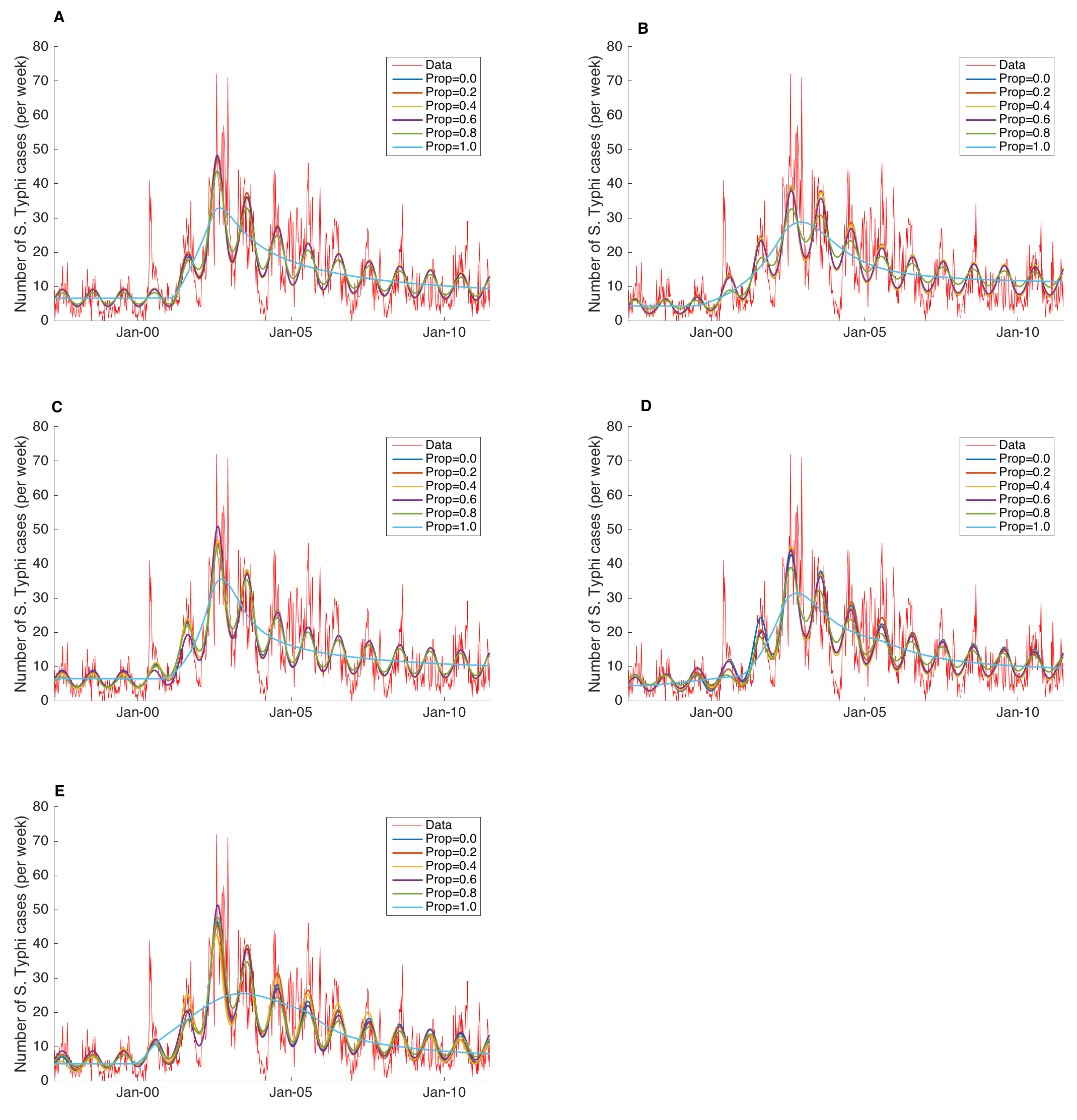

Supplement: S2 Fig — (A) Scenario 1; (B) Scenario 2; (C) Scenario 3; (D) Scenario 4; (E) Scenario 5. Observed weekly cases of S. Typhi (red); best-fit model with 0% R0p and 100% R0w transmission (navy); best-fit model with 20% R0p and 80% R0w transmission (magenta); best-fit model with 40% R0p and 60% R0w transmission (yellow); best-fit model with 60% R0p and 40% R0w transmission (purple); best-fit model with 80% R0p and 20% R0w transmission (green); best-fit model with 100% R0p and 0% R0w transmission (light-blue). (TIFF) [file pntd.0005547.s003.tiff]

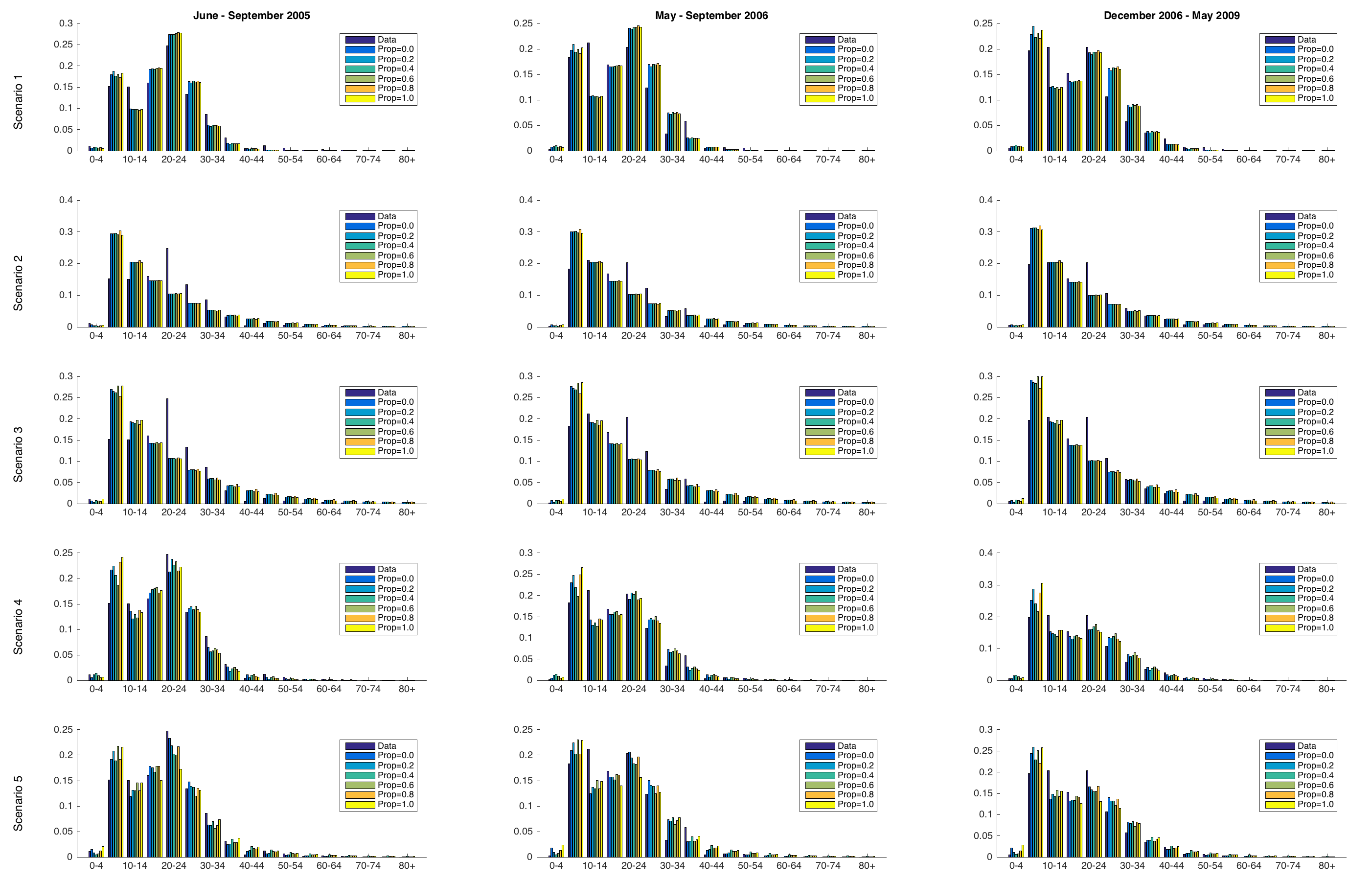

Supplement: S3 Fig — Observed and model-predicted age distribution shown for the three time periods for which age-specific data on cases was available. Columns represent the age distribution for a given time period while the rows indicate the age distribution for a given model scenario. (TIFF) [file pntd.0005547.s004.tiff]

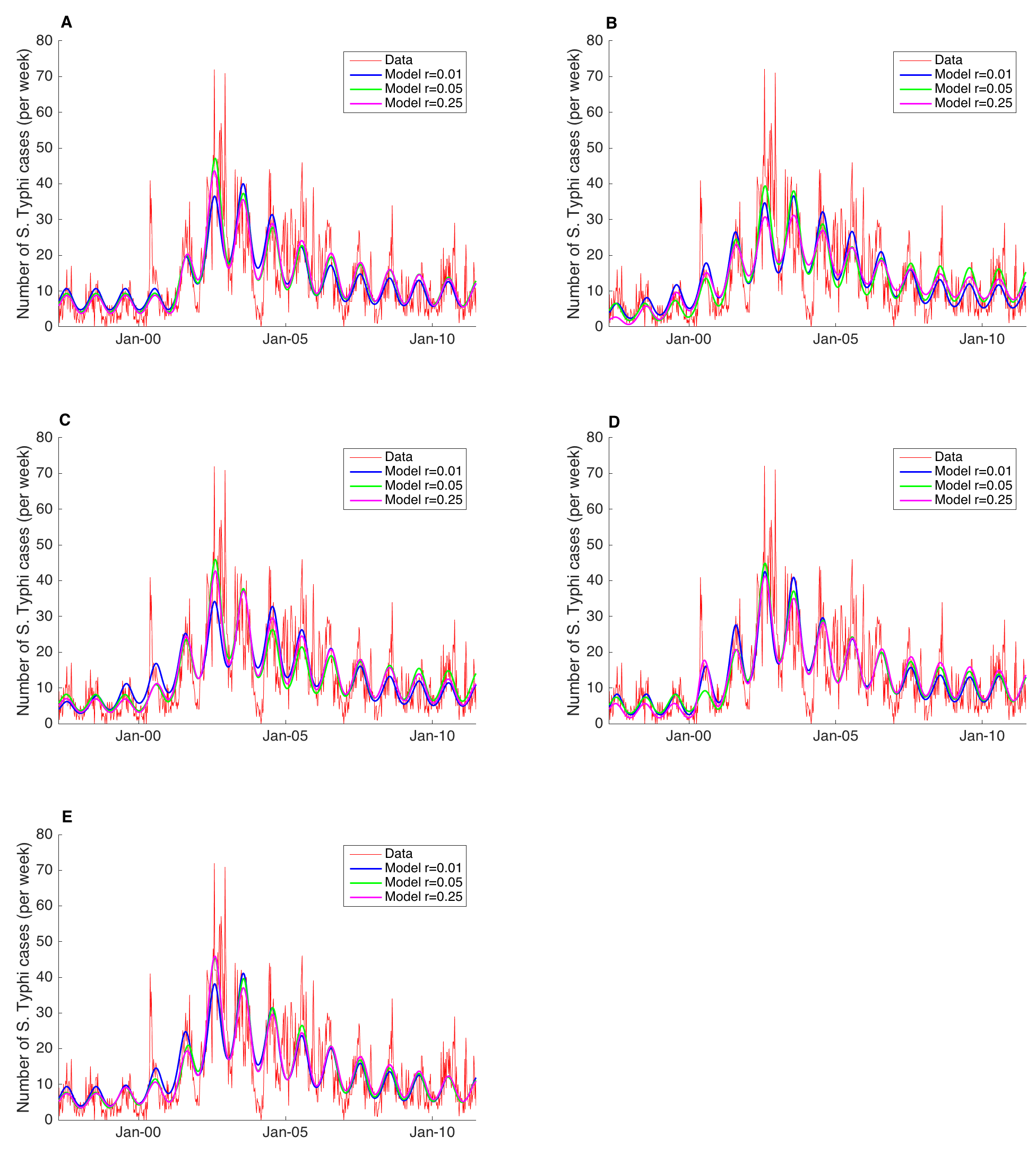

Supplement: S4 Fig — (A) Scenario 1; (B) Scenario 2; (C) Scenario 3; (D) Scenario 4; (E) Scenario 5. Observed weekly cases of S. Typhi (red); best-fit model with r = 0.01 (blue); best-fit model with r = 0.05 (green); best-fit model with r = 0.25 (magenta). (TIFF) [file pntd.0005547.s005.tiff]

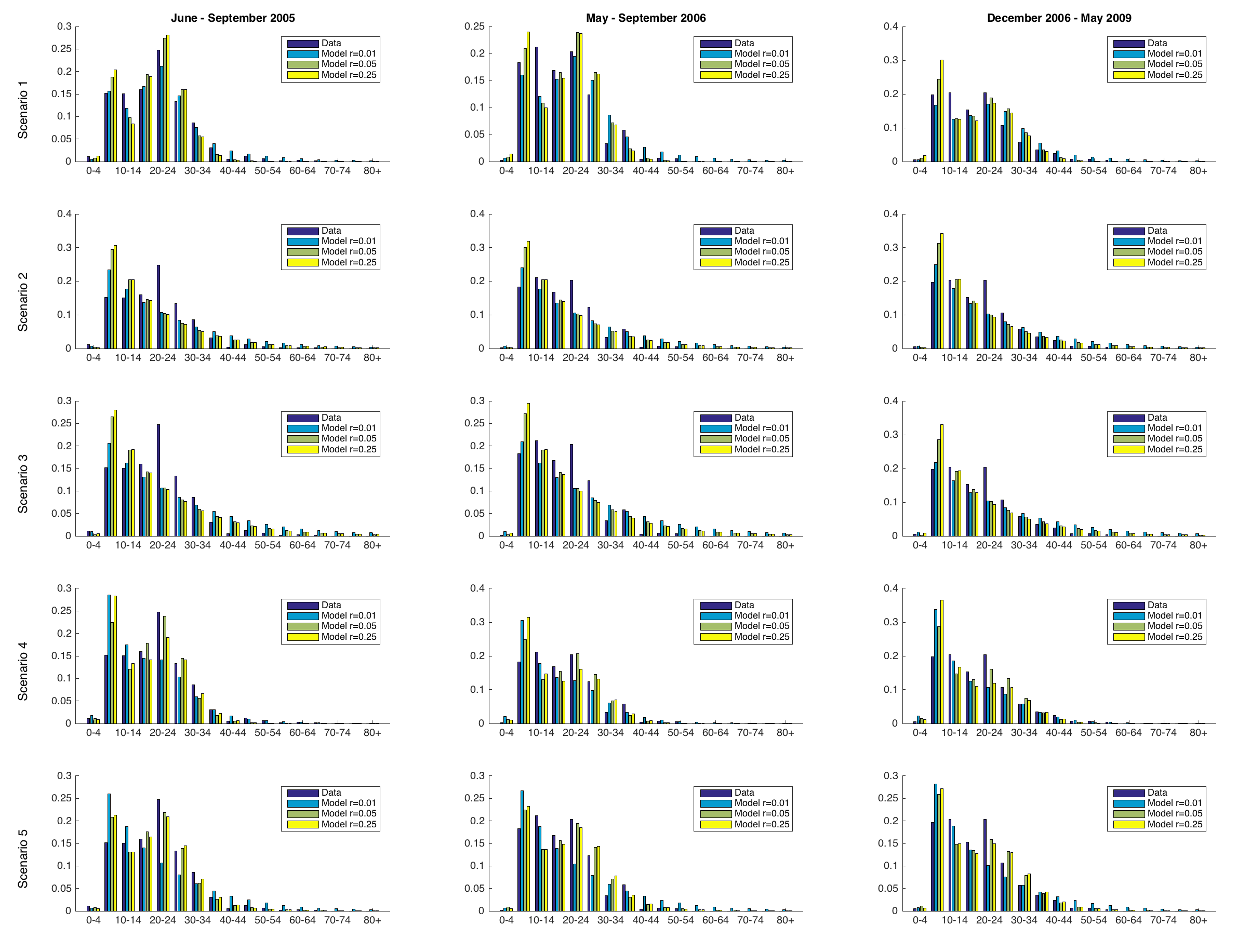

Supplement: S5 Fig — Observed and model-predicted age distribution shown for the three time periods for which age-specific data on cases was available. Columns represent the age distribution for a given time period while the rows indicate the age distribution for a given model scenario. (TIFF) [file pntd.0005547.s006.tiff]

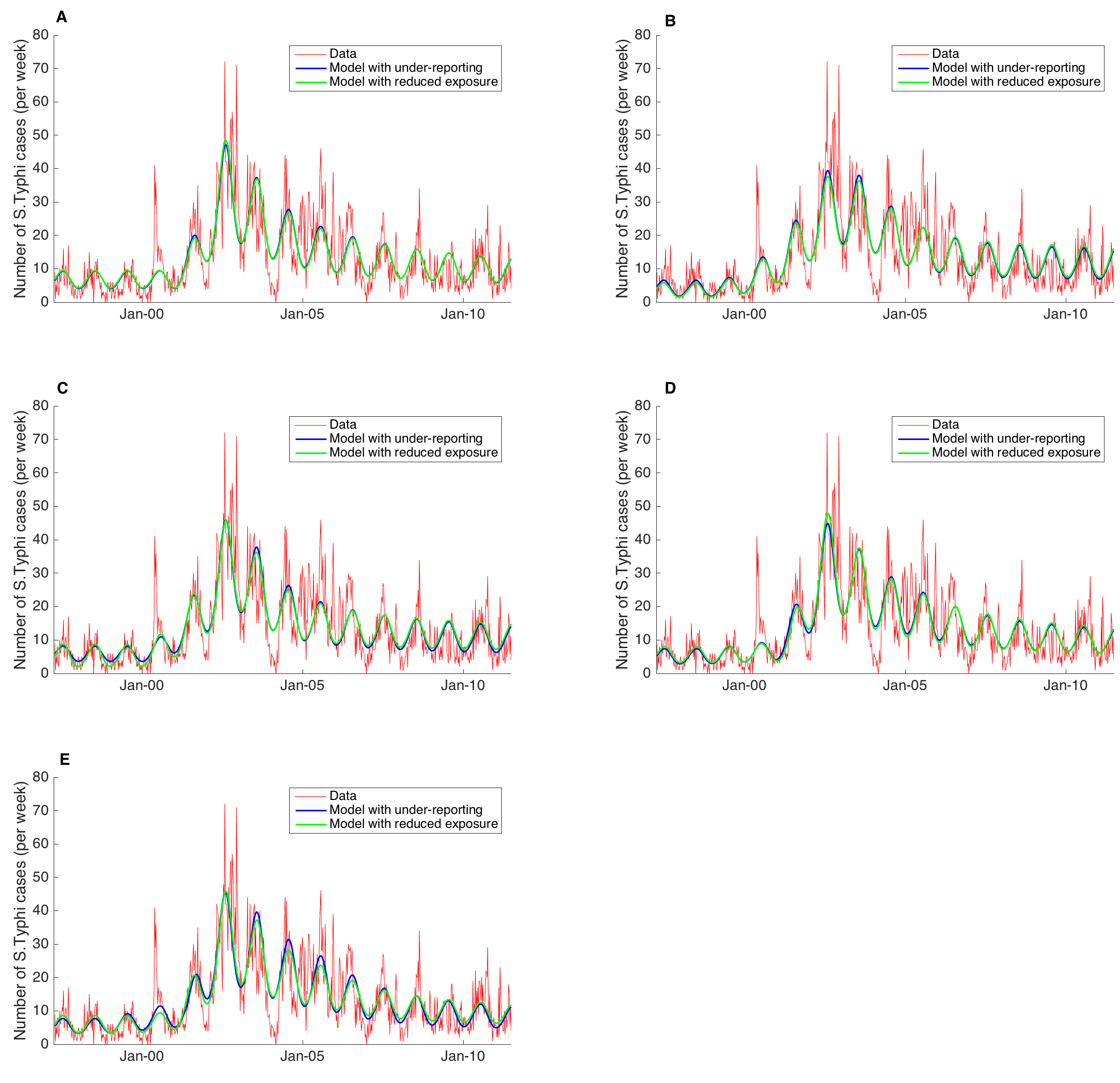

Supplement: S6 Fig — (A) Scenario 1; (B) Scenario 2; (C) Scenario 3; (D) Scenario 4; (E) Scenario 5. Observed weekly cases of S. Typhi (red); best-fit model with under-reporting (blue); best-fit model with reduced exposure (green). (TIFF) [file pntd.0005547.s007.tiff]

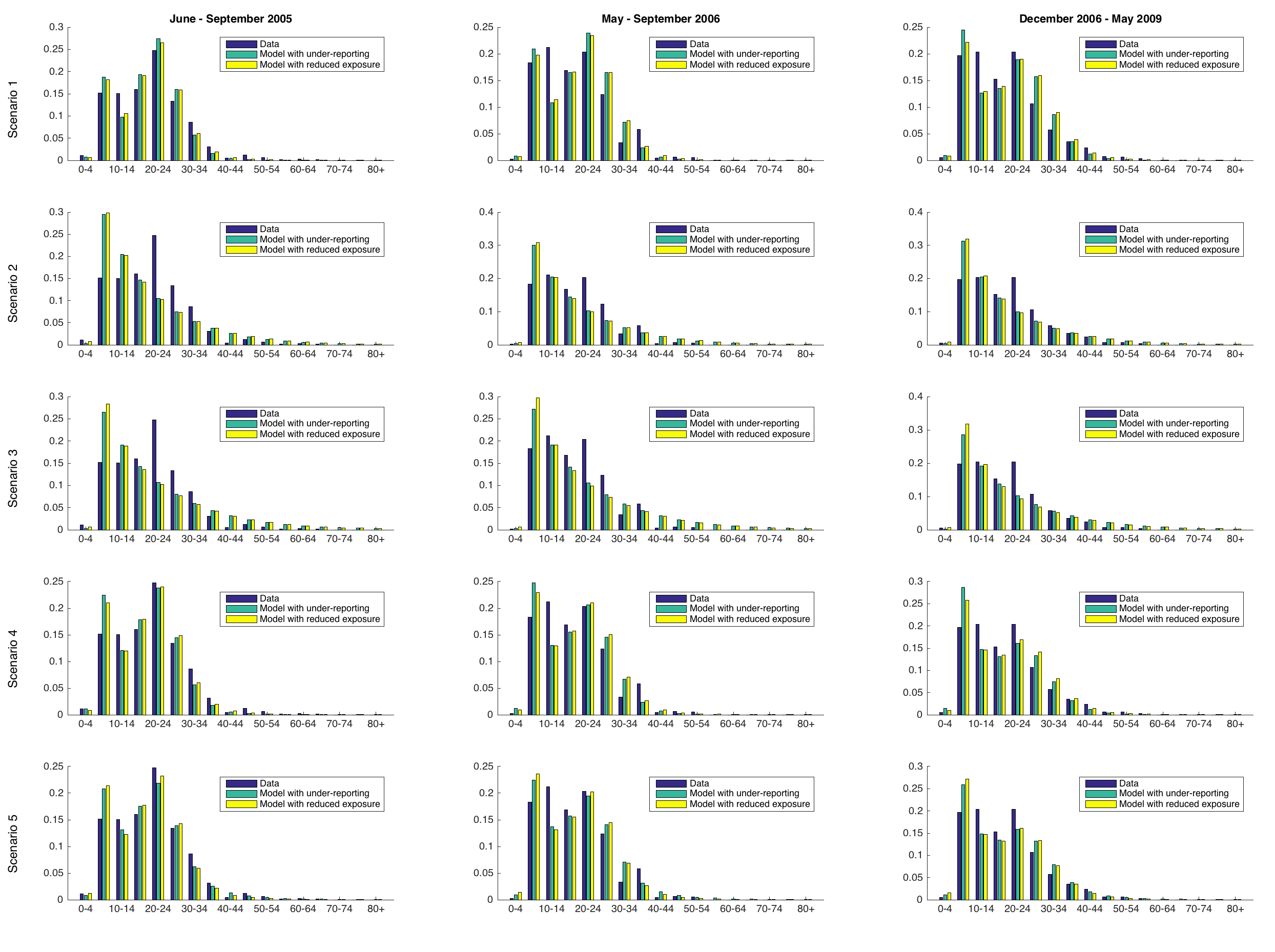

Supplement: S7 Fig — Observed and model-predicted age distribution shown for the three time periods for which age-specific data on cases was available. Columns represent the age distribution for a given time period while the rows indicate the age distribution for a given model scenario. (TIFF) [file pntd.0005547.s008.tiff]
